# Development and Validation of a Web-Based Survey on the Use of Personal Communication Devices by Hospital Registered Nurses: Pilot Study

**DOI:** 10.2196/resprot.2774

**Published:** 2013-11-26

**Authors:** Deborah L McBride, Sandra A LeVasseur, Dongmei Li

**Affiliations:** ^1^School of Nursing and Dental HygieneUniversity of Hawaii at ManoaHonolulu, HIUnited States; ^2^Hawaii State Center for NursingSchool of Nursing and Dental HygieneUniversity of HawaiiManoa, HIUnited States; ^3^Department of Public Health SciencesOffice of Public Health StudiesUniversity of HawaiiManoa, HIUnited States

**Keywords:** cellular phones, Internet, electronic mail, text messaging, nurses, distraction, medical staff/psychology, attitude of health personnel, survey methodology, questionnaire design

## Abstract

**Background:**

The use of personal communication devices (such as basic cell phones, enhanced cell phones or smartphones, and tablet computers) in hospital units has risen dramatically in recent years. The use of these devices for personal and professional activities can be beneficial, but also has the potential to negatively affect patient care, as clinicians may become distracted by these devices.

**Objective:**

No validated questionnaire examining the impact of the use of these devices on patient care exists; thus, we aim to develop and validate an online questionnaire for surveying the views of registered nurses with experience of working in hospitals regarding the impact of the use of personal communication devices on hospital units.

**Methods:**

A 50-item, four-domain questionnaire on the views of registered nursing staff regarding the impact of personal communication devices on hospital units was developed based on a literature review and interviews with such nurses. A repeated measures pilot study was conducted to examine the psychometrics of a survey questionnaire and the feasibility of conducting a larger study. Psychometric testing of the questionnaire included examining internal consistency reliability and test-retest reliability in a sample of 50 registered nurses.

**Results:**

The response rate for the repeated measures was 30%. Cronbach coefficient alpha was used to examine the internal consistency and reliability, and in three of the four question groups (utilization, impact, and opinions), the correlation was observed to be very high. This suggests that the questions were measuring a single underlying theme. The Cronbach alpha value for the questions in the performance group, describing the use of personal communication devices while working, was lower than those for the other question groups. These values may be an indication that the assumptions underlying the Cronbach alpha calculation may have been violated for this group of questions. A Spearman rho correlation was used to determine the test-retest reliability. There was a strong test-retest reliability between the two tests for the majority of the questions. The average test-retest percent of agreement for the Likert scale responses was 74% (range 43-100%). Accounting for responses within the 1 SD range on the Likert scale increased the agreement to 96% (range 87-100%). Missing data were in the range of 0 to 7%.

**Conclusions:**

The psychometrics of the questionnaire showed good to fair levels of internal consistency and test-retest reliability. The pilot study demonstrated that our questionnaire may be useful in exploring registered nurses’ perceptions of the impact of personal electronic devices on hospital units in a larger study.

## Introduction

The use of personal communication devices including Internet surfing, text messaging, and emailing has increased in recent years. Electronic tools such as basic cell phones, enhanced cell phones (smartphones), and tablet computers are becoming mainstream, and numerous positive benefits of personal communication devices are cited in the literature. They provide clinicians with rapid access to medical references and patient information [1]. They are used for medical consultation [2], documentation [3], and patient education [4], and applications have been created for many clinical specialties [5-8].

There is much debate about whether online distractions, regardless of whether they are personal or professional in nature, can prove hazardous in medical settings, potentially distracting health care workers from patient care (termed “distracted nursing”) [9]. For example, the Joint Commission for the Accreditation Organization [10] and the United States Pharmacopeia through MEDMARX analyzed medication error reports and found that 43% of medication errors in hospitals were attributable to workplace distractions [11]. The ECRI Institute, a nonprofit health care research organization, publishes an annual top 10 technology hazards list. The ninth hazard on this list for 2013 was “Caregiver distractions from smartphones and other mobile devices” [9]. Concern among surgeons about distraction caused by the use of cellular telephone technology in the operating room led the American College of Surgeons to issue a statement (ST-59). The statement says, “the undisciplined use of cellular devices in the [operating room]—whether for telephone, e-mail, or data communication, and whether by the surgeon or by other members of the surgical team—may pose a distraction and may compromise patient care” [12].

Rapidly increasing technology fosters multitasking because it promotes multiple sources of input. A key concern about such multitasking is that this increase in simultaneous media consumption decreases the amount of attention paid to each device [13,14].

Several studies have reported the effects of using personal communication devices while driving [15]. It has been reported that drivers react more slowly to brake lights and stop signs during phone conversations [16], and additional driving studies have shown that peripheral vision is reduced when using a cell phone [17]. These studies have led to numerous new state laws prohibiting the use of personal communication devices for calling, texting, or both while driving.

Based on this research and findings that interruptions to patient care are common in hospitals [18] and may contribute to errors in said care, we chose to initiate a study into how “distracted nursing” might affect patient care in a hospital setting. For the purpose of this research, we propose the development and validation of an online survey to identify the concerns and opinions of registered nurses who had experience of working in hospitals regarding the effects of using personal communication devices while on duty in hospital units. This paper sets out the process for the development and initial testing of an online survey. We hypothesize that, in the future, the information obtained from this survey could be used to determine policies for the use of personal communication devices on hospital units to minimize risks to patients.

## Methods

### Questionnaire Research and Development Process

#### Domain Identification

In our literature review, we searched the Cumulative Index to Nursing and Allied Health, PubMed, and Dissertation Abstractions International databases for relevant publications on distraction and communication devices related to health care workers in the 2003-2012 period. As the term “cellular phone” was first introduced as a MeSH keyword in 2003, we selected this year as the starting point. The search terms for the present research were as follows: “cellular phones”, “Internet”, “computers, portable”, “electronic mail”, “text messaging”, “nurses”, “healthcare workers”, “distraction”, “medical staff/psychology”, and “attitude of health personnel”.

#### Item Development

We reviewed previous questionnaires used in surveys related to distraction and personal communication devices and included the following as items in our survey: opinions about how cell phones impact team effectiveness [19], registered nurses’ opinions about cell phone use and patient safety [20], and frequency of Internet use for personal activities at work [21].

A total of 64 potential survey items were identified based on criteria outlined by DeVellis [22]. These survey items were reformulated, assessed, refined, or rejected in collaboration with a nursing team with knowledge about questionnaire methodology, consisting of 3 faculty members at nursing schools, all of whom had worked as nurses for more than 20 years. The items were also discussed with a unit manager and 2 hospital nurses, all of whom were certified clinical nurse specialists and had worked for more than 10 years on their nursing units.

#### Format of the Questionnaire

The 50-item pilot questionnaire adhered to the recommendations of Bowling [23]. The first item in the questionnaire asked respondents to choose a 4-digit identification number that they would be asked to provide again in the second survey to assist us in matching their test-retest surveys while remaining anonymous.

A total of 10 survey items used a drop-down menu asking respondents to select one answer, including the demographics: gender, age, race/ethnicity, type of workplace setting, and primary nursing practice position. The demographic questions were developed in accordance with the Forum of State Nursing Workforce Centers for Standardizing Nursing Workforce Data [24].

There were 2 types of questions following the initial demographic questions. The first type aimed to gain straightforward numerical-type data through the use of three different 4-point Likert scales (1) “strongly disagree”, “disagree”, “agree”, and “strongly agree”; (2) “>5 times”, “2-5 times”, “once”, and “never”; and (3) “strongly negative”, “slightly negative”, “slightly positive”, and “strongly positive”. No scale had a “don’t know” option. The second type of question probed the reasoning behind why respondents had answered specific Likert-scale questions (“If yes, please describe”) or asked them to state recommendations.

The other 5 items using drop-down menus, where respondents were asked to select one answer, sought their opinion of personal communication device use while at work (2 items), hospital policy concerning the use of device in hospital units, how concerned their employer is about nonwork-related use of personal communication devices, and their awareness of their employer disciplining or terminating the employment of a nurse for excessive personal communication device use while working.

The survey then included a total of 32 Likert-type questions that consisted of: 14 questions on personal communication device use on hospital units; 9 questions on the effects of these devices on job performance, and specifically, how they affect patient care; 6 questions on how the devices influence coordination and teamwork; and 3 questions on how nurses think patients or other health care providers perceive a nurse using their personal communication device during work time.

Following the Likert-type questions, there were 7 open-ended questions. One question asked respondents to provide a description of their hospitals’ personal communication device policy. Another one queried respondents’ opinions about how these devices should be used in hospital units. Respondents were asked in another question to address the overall impact of these devices on nursing units. Two of the questions asked for examples of how personal communication devices had positively or negatively affected a nurse’s performance. One question queried respondents if they were aware of a situation where their employer had disciplined or terminated the employment of anyone for excessive use of a personal communication device at work and, if so, to describe the situation. The last question asked respondents for any additional comments about the use of personal communication device in hospital units.

### Validation Process

**Figure 1:**
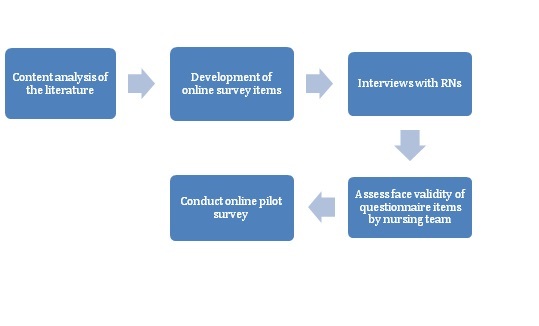
The validation process for the development of the online survey.

The validation process is set out in Figure 1, and was based on criteria proposed by Terwee et al [25]. First, the items for inclusion were developed from a content analysis of the literature as described above. Informal interviews with a small convenience sample of registered nurses using personal communication devices on general medical-surgical units took place to identify themes relevant to nurses working in clinical settings. A team of 3 nursing experts assessed the initial 64 items selected for face validity and content validity [26]. Finally, the number of items was reduced to 50 for the pilot online questionnaire.

### Online Pilot Survey

Following the validity testing, an online pilot survey was conducted in November 2012, with 50 registered nurses associated with acute care settings in Honolulu, Hawaii and the San Francisco Bay area in California. Only registered nurses who had worked in a hospital unit for at least 5 months in the previous 2 years were eligible to participate in the survey.

For the pilot online survey, two versions of the same questionnaire were created with questions randomly arranged within blocks of grouped questions. The registered nurses were invited to participate in the survey and asked to complete two separate online questionnaires, accessed via a hyperlink email sent 1 week apart. The interval between the two questionnaires was set for 1 week because it limits the recall of responses but does not generally allow enough time for respondents to have altered their attitudes or behaviors [21,26]. The random arrangement of questions within each block was to check that there was no order effect bias [27].

No literature was found to have similar study settings to our pilot project. A sample size could not be calculated from power analysis, but a sample size of 12 per group is a useful principle based on experience, according to Julius [28]. A sample of 50 registered nurses was chosen for the pilot study considering a conservative 50% nonresponse rate. We planned to use data collected from the pilot study to determine power and sample size for a larger online survey.

### Data Analysis

The pilot online survey data were downloaded to Excel (version 14.2.5, Microsoft Corporation), and statistical analyses were performed using SPSS version 21 (SPSS Inc). Descriptive statistics were used to examine missing data and the floor and ceiling effects [26]. Spearman correlation coefficients were used to examine test-retest reliability for agreement with the level of significance set at *P*<.05. Cronbach alpha was used to examine the grouped question items for internal consistency reliability and kappa scores to measure reliability.

The online questionnaire comments were analyzed using the meaning condensation method introduced by Kvale and Brinkmann [29] in which the main sense of what is said is rephrased into more succinct formulations, and content analysis [30].

### Ethics

Based on the Code of Federal Regulations 45 CFR 46 (2), this online pilot study was exempt from institutional review board approval. Permission to conduct the exempt study was granted by the University of Hawaii Human Subjects Committee on September 13, 2012. All participants were informed in writing of the purpose of the study, that participation was voluntary, and that responses were anonymous.

## Results

### Respondents

The overall response rate for the pilot survey is shown in Table 1. Of the 50 invited respondents, 15 (30%) respondents completed both the test and retest online surveys. Table 1 also presents background information of the participants in the pilot surveys. The mean age of the 15 respondents answering both online surveys was 49.23 (SD 10.2) years. Respondents who completed both surveys were primarily female 13/15 (87%), and 35/50 (70%) participants were missing/nonresponders.

### Face and Content Validity

Based on the responses to the open-ended questions, the respondents in the pilot online survey understood the questions. Respondents considered the questions relevant, and none found areas lacking. In response to discussions with experienced colleagues in nursing research methodology, we made minor adjustments to phrasing and response options in multiple-response questions.

### Reliability and Agreement

In the pilot survey, 19/32 responses (59%) to the 4-point Likert-scale questions covered all response categories. However, the variability in answers was not spread evenly across all questions. Test-retest reliability for respondents who completed both questionnaires was determined by intrarespondent percent agreement for each of the Likert-type questions. The results indicate that 74% (range 43-100%) of the answers to the questions were the same for both questionnaires (Tables 2-5); accounting for responses within the 1 SD range on the Likert scale increased the agreement to 96% (range 87-100%).

Cohen’s kappa was calculated for each Likert-scale question on the online test/retest pilot survey to assess the items’ inter-rater agreement. Comparing the answers from the two versions of the questionnaire, the mean Cohen’s kappa for the Likert-scale questions was .41 (41%, range .67 to −.057). A kappa of 1 indicates perfect agreement, whereas a kappa of 0 indicates agreement equivalent to chance [31].

The discrepancy between the relatively low kappa results and the high percent of agreement is most likely due to the sensitivity of kappa to small sample sizes (n=15). It is also possible that the registered nurses undergoing the survey changed their attitudes during the 1-week interval between the two questionnaires. Such a shift could have been subtle, but any difference results in a lower kappa value.

**Table 1:** Respondent demographics in the first, second, or paired test-retest online surveys (N=50).

		First survey, n (%)^a^	Second survey, n (%)^a^	Paired surveys, n (%)^a^
**Gender**				
	Total respondents	39 (78)	21 (42)	15 (30)
	Male	5 (10)	2 (4)	2 (4)
	Female	34 (68)	19 (38)	13 (26)
	Missing; nonrespondents	11 (22)	29 (58)	35 (70)
**Age, years**				
	Total respondents	39 (78)	21 (42)	15 (30)
	<30	1 (2)	0 (0)	0 (0)
	30-40	5 (10)	3 (6)	3 (6)
	40-50	8 (16)	5 (10)	4 (8)
	50-60	12 (24)	8 (16)	6 (12)
	>60	13 (26)	5 (10)	2 (4)
	Missing; nonrespondents	11 (22)	29 (58)	35 (70)
**Race/ethnicity**				
	Total respondents	38 (76)	21 (42)	15 (30)
	American Indian or Alaska Native	0 (0)	1 (2)	1 (2)
	Asian	1 (2)	1 (2)	1 (2)
	Black/African American	0 (0)	0 (0)	0 (0)
	Native Hawaiian/Pacific Islander	2 (4)	0 (0)	1 (2)
	White/Caucasian	31 (62)	19 (38)	12 (24)
	Hispanic/Latino	1 (2)	0 (0)	0 (0)
	Other	3 (6)	0 (0)	0 (0)
	Missing; nonrespondents	12 (24)	29 (58)	35 (70)
**Workplace setting**				
	Total respondents	39 (78)	21 (42)	15 (30)
	Hospital	17 (34)	9 (18)	8 (16)
	Nursing home/extended care	1 (2)	1 (2)	1 (2)
	Home health	1 (2)	0 (0)	0 (0)
	Academic setting	16 (32)	6 (12)	3 (6)
	Ambulatory care setting	1 (2)	0 (0)	0 (0)
	Insurance claims/benefits	1 (2)	1 (2)	1 (2)
	Other	2 (4)	3 (6)	2 (4)
	Missing; nonrespondents	11 (22)	29 (58)	35 (70)
**Position title**				
	Total respondents	39 (78)	21 (42)	15 (30)
	Consultant/nurse researcher	1 (2)	1 (2)	1 (2)
	Nurse executive	0 (0)	2 (4)	0 (0)
	Nurse unit manager	3 (6)	2 (4)	1 (2)
	Nurse faculty	17 (34)	7 (14)	4 (8)
	Advanced practice nurse	2 (4)	0 (0)	1 (2)
	Staff nurse	13 (26)	7 (14)	6 (12)
	Charge nurse	1 (2)	1 (2)	1 (2)
	Other—health related	1 (2)	1 (2)	1 (2)
	Other—not health related	1 (2)	0 (0)	0 (0)
	Missing; nonrespondents	11 (22)	29 (58)	35 (70)
**Personal communication device (PCD)**				
	Total respondents	30 (60)	19 (38)	15 (30)
	Do not have a PCD	2 (4)	1 (2)	1 (2)
	Basic PCD (cell phone only)	4 (8)	2 (4)	2 (4)
	PCD (cell phone and texting)	3 (6)	3 (6)	3 (6)
	PCD (cell phone/texting/Internet/email/apps)	21 (42)	13 (26)	9 (18)
	Tablet computer	0 (0)	0 (0)	0 (0)
	Missing; nonrespondents	20 (40)	31 (62)	35 (70)

^a^Percentages may not sum due to rounding.

**Table 2:** Respondents with the same answers to both online pilot surveys for Group 1: utilization scale.

Please select the column that best describes your opinion about the use of personal communication devices by nurses at work.	Never, n (%)	Once per day, n (%)	2-5 times per day, n (%)	>5 times per day, n (%)	Spearman rho	*P*
I access work-related drug references.	8 (53)	1 (7)	0 (0)	0 (0)	.862^b^	<.001
I access work-related nursing/medical information.	6 (40)	3 (20)	2 (13)	0 (0)	.750^b^	.001
I use the device as a calculator for nursing/medical formulas.	4 (40)	2 (13)	0 (0)	1 (7)	.858^b^	<.001
I access work-related protocols.	7 (47)	3 (20)	0 (0)	0 (0)	.605^a^	.02
I access work-related apps that assist patient care.	9 (60)	2 (13)	1 (7)	0 (0)	.603^a^	.02
I access sites for professional education and development.	6 (43)	2 (14)	0 (0)	0 (0)	.603^a^	.02
I access sites for patient handouts and teaching.	10 (71)	1 (7)	0 (0)	0 (0)	.994^b^	<.001
I use it to communicate with other members of the health care team to coordinate patient care.	7 (50)	2 (14)	2 (14)	0 (0)	.914^b^	<.001
I check/send personal text messages or emails to family or friends.	4 (27)	2 (13)	2 (13)	0 (0)	.638^a^	.01
I shop on the Internet.	13 (87)	0 (0)	0 (0)	0 (0)	−.071	.80
I check/post on social networking sites (Facebook, Twitter, etc) (excluded from analysis because of lack of variation in responses).	14 (93)	0 (0)	0 (0)	0 (0)	-^c^	-^c^
I play online games.	12 (86)	0 (0)	0 (0)	0 (0)	.734^b^	.003
I check/send nonwork-related text messages or emails to coworkers.	6 (43)	0 (0)	0 (0)	0 (0)	.386	.17
I conduct personal business online (eg, paying bills, banking).	13 (87)	1 (7)	0 (0)	0 (0)	.681^b^	.005

^a^Correlation is significant at the .05 level (2-tailed).

^b^Correlation is significant at the .01 level (2-tailed).

^c^Cannot be computed because at least one of the variables is constant.

**Table 3:** Respondents with the same answers to both online pilot surveys for Group 2: performance scale.

Please select the column that best describes use of your personal communication device while working (excluding breaks or meal times).	Never, n (%)	Once per shift, n (%)	2-5 times per shift, n (%)	> 5 times per shift, n (%)	Spearman rho, n (%)	*P*
My personal communication device for nonwork-related activities has distracted me (reversed scale).	8 (53)	3 (20)	0 (0)	0 (0)	.443	.11
My personal communication device for nonwork-related activities has negatively affected my performance (reversed scale).	10 (77)	1 (7)	0 (0)	0 (0)	.362	.22
I have witnessed nurses whose personal communication devices have negatively affected their performance (reversed scale).	4 (29)	5 (36)	0 (0)	1 (7)	.704^a^	.005
My personal communication device for nonwork-related activities has helped me focus on my work.	6 (43)	4 (29)	0 (0)	0 (0)	.463	.10
My personal communication device for nonwork-related activities has positively affected my performance.	7 (50)	5 (36)	0 (0)	0 (0)	.708^a^	.005
I have witnessed nurses whose personal communication devices have positively affected their performance.	6 (46)	4 (31)	1 (7)	0 (0)	.750^a^	.003
I have made a medical error that was a result of distraction caused by use of my personal communication device (excluded because of lack of variation in responses).	13 (93)	0 (0)	0 (0)	0 (0)	-^b^	-^b^
I have witnessed another nurse making a medical error that was the result of distraction caused by his/her use of a personal communication device (excluded to lack of variation in responses).	14 (93)	0 (0)	0 (0)	0 (0)	-^b^	-^b^
I am aware of a serious medical accident that was the result of a nurse being distracted while using his/her personal communication device (excluded because of lack of variation in response).	14 (100)	0 (0)	0 (0)	0 (0)	-^b^	-^b^

^a^Correlation is significant at the .01 level (2-tailed).

^b^Cannot be computed because at least one of the variables is constant.

**Table 4:** Respondents with the same answers to both online pilot surveys for Group 3: impact scale.

Please select the column that best describes your opinion about nurses’ use of personal communication devices at work.	Strongly disagree, n (%)	Disagree, n (%)	Agree, n (%)	Strongly agree, n (%)	Spearman rho	*P*
Use of personal communication devices in nursing units has enabled better coordinated patient care among nursing/medical teams.	3 (21)	3 (21)	4 (29)	0 (0)	.411	.15
Use of personal communication devices in the nursing unit improves unit cohesion and teamwork.	3 (21)	3 (21)	4 (29)	0 (0)	.059^a^	.02
Use of personal communication devices in the nursing unit improves patient safety.	2 (14)	2 (14)	4 (29)	0 (0)	.468	.11
Personal communication devices in the nursing unit are beneficial to patient care.	1 (7)	1 (7)	4 (29)	0 (0)	.161	.60
Use of personal communication devices at work helps me resolve personal issues quickly and improves my ability to focus on work.	2 (14)	2 (14)	2 (14)	0 (0)	.283	.33
Use of personal communication devices at work reduces work-related stress and improves patient care.	2 (14)	1 (7)	3 (21)	0 (0)	.489	.09

^a^Correlation is significant at the .05 level (2-tailed).

**Table 5:** Respondents with the same answers to both online pilot surveys for Group 4: opinion scale.

Please select the column that best describes your opinion about nurses’ use of personal communication devices at work.		Strongly negative, n (%)	Negative, n (%)	Positive, n (%)	Strongly positive, n (%)	Spearman rho	*P*
How do you feel about nurses when you see them using their personal communication devices on the unit?		3 (21)	4 (29)	1 (7)	0 (0)	.648^a^	.01
How to you think patients feel when they see a nurse using his/her personal communication device on the unit?		6 (43)	5 (36)	0 (0)	0 (0)	.645^a^	.01
How do you think other health care staff feel when they see a nurse using his/her personal communication device on the unit?		4 (29)	8 (57)	0 (0)	0 (0)	.073^b^	.003

^a^Correlation is significant at the .05 level (2-tailed).

^b^Correlation is significant at the .01 level (2-tailed).

### Internal Consistency

For the purposes of measuring survey reliability using Cronbach alpha, the survey was organized into 4 groups: (1) utilization of personal communication devices in nursing units, (2) effects of personal communication devices on registered nurses’ performance, (3) registered nurses’ opinions about personal communication device use and patient safety, and (4) registered nurses’ knowledge about hospital policy concerning personal communication device use in hospital units.

Several modifications were required to Group 2 questions. The final 3 questions (see Table 3, Group 2 questions) were excluded from the reliability analysis because nearly all respondents gave a response of “Never” to these questions. This lack of variation in the responses made it impossible to calculate reliable statistics, so the analysis used only the 6 remaining questions from Group 2. Of the 6 remaining questions, 3 dealt with negative events, whereas the other 3 dealt with positive events. To ensure consistency in the interpretation of the responses, the response scale was reversed for the 3 negative questions so that when calculating reliability statistics for this group, a positive response to a negative question was counted the same way as a negative response to a positive question. The 7 open-ended questions were excluded from the reliability analysis, as they did not share a common scale with the other questions.

### Reliability of Grouped Questions

Cronbach alpha (ranging between 0 and 1) was selected as the most appropriate statistical analysis for testing the reliability of grouped questions because it can be used on ordinal data, such as the Likert scale. Higher alpha values imply a greater degree of correlation or inter-relatedness, with .7 generally considered a minimally acceptable value for reliability [31].

Table 6 shows the Cronbach alpha statistic for each of the four sets of grouped questions. For each survey, two analyses of the datasets were run: (1) inclusion of all survey respondents (ie, includes respondents who completed only one questionnaire), and (2) inclusion of only those respondents who completed both questionnaires. The unpaired alpha values make use of all of the available data to produce a reliability estimate based on as many responses as possible, but may be susceptible to selection bias if there is a systematic pattern in the types of respondents who completed both surveys. The paired values eliminate this concern by ensuring that the same set of respondents is included in both Week 1 and Week 2 calculations, but sacrifice sample size to achieve this. Before computing these reliability statistics, all the responses to these questions were converted to a simple number scale, assigning integer values to each response (“never”, “strongly disagree”, or “strongly negative” was converted to 1; “once a day”, “disagree”, or “slightly negative” was converted to 2; “2-5 times per day”, “agree”, or “slightly negative” was converted to 3; “>5 times per day”, “strongly agree”, or “strongly positive” was converted to 4). No attempt was made to quantify the answers or the gaps between them accurately; the scale was kept simple for convenience.

Table 6 shows that the measured reliability of 3 of the 4 question groups (utilization, impact, and opinions) was high, indicating that responses in these groups tended to be highly correlated. This suggests that the questions were measuring a single underlying theme. For these 3 groups, the reliability remained high in both weeks, and there was little variation based on whether all responses or only paired responses were included.

The Cronbach alpha value for Group 2 performance questions, describing use of personal communication devices by registered nurses while working, was notably lower than that for other question groups. The Week 2 values indicated that responses were fairly consistent, but the Week 1 responses displayed a very low Cronbach alpha value of .08, or −.35 if only paired responses were examined.

Because of the lack of variation in responses, 3 questions from Group 2, related to medical errors, and 1 question from Group 1, which asked respondents if they posted on social networking sites while working, were excluded from the survey.

**Table 6:** Cronbach alpha for grouped survey questions.

Question group	Week 1		Week 2	
	All responses	Paired responses	All responses	Paired responses
Group 1: Utilization	.83	.76	.92	.84
Group 2: Performance	.08	−.35	.63	.45
Group 3: Impact	.89	.96	.97	.96
Group 4: Opinion	.82	.89	.89	.85

### Floor and Ceiling Effect

The floor and ceiling effect is detected using the measures of central tendency of the data, including mean and median, as well as the range, SD, and skewness [24]; a score would be considered acceptable if the values are distributed in a normal or bell-shaped curve, with the mean near the midpoint of the scale. Some criteria for floor and ceiling effects recommend a skewness statistic between −1 and +l as acceptable for eliminating the possibility of these effects.

Responses to the Likert scales revealed that 16/32 (50%) questions on the pilot online survey had a ceiling effect (ie, higher than the recommended maximum of 15%). However, given the small sample size of the pilot online survey, floor/ceiling effect criteria could not be applied to the survey results.

### Open-Ended Questions

A total of 33/50 respondents (66%) from the pilot study added comments (some example comments are included in Table 7).

**Table 7:** Comments provided in response to open-ended questions in online pilot surveys.

Question	Comment
If you have witnessed the use of personal communication devices negatively affecting either your or another nurse’s performance, please describe here.	I have witnessed nurses’ cell phones ringing while at the patient’s bedside. The nurse answers it and walks away from the patient. I have also witnessed nurses staying at the nurses’ station on their devices.
If you have witnessed the use of personal communication devices positively affecting either your or another nurse’s performance, please describe here.	I receive one text message per day when my husband leaves his night shift work at the jail. I know he is safe and I can concentrate on my work.
Are you aware of your employer disciplining or terminating a nurse employee for excessive use of his/her personal communication device for nonwork-related activities while working? If yes, please describe here.	Staff in Labor and Delivery were on Facebook. Management declared that these employees were using employer’s assets during work time.
Please describe how you think an employer should handle the use of personal communication devices at work.	Ringer must be put into vibrate mode only. Personal communications can be made only during breaks. Use for patient-related care should be encouraged.

### Responsiveness

Responses to the online pilot survey were segmented by demographic variables (eg, gender, ethnicity, age, and job title). We performed regression analyses to determine if any divergent attitudes and/or behaviors based on demographics were statistically significant. None were statistically significant because of the small sample size.

### Missing Data

In the online pilot survey, the first dataset had an average percentage of missing data of 1.2% and the second dataset of 0.04%.

In the paired composite data, there were 35 missing answers, resulting in 0.03% missing data. However, only one respondent was responsible for 17/35 missing answers (48%) in the paired composite data. Eliminating this respondent reduced the sample size to 14 pairs and the percent of missing data to 0.01%. In analyzing the data, a listwise deletion was used because the percentage of missing data was small (<5%) and the listwise deletion has been shown to provide unbiased estimators [32].

## Discussion

### Principal Findings

The present study describes a validation process to examine the psychometrics of a newly developed questionnaire concerning the views of registered nurses on the impact of the use of personal communication devices on nursing units. It concludes that initial findings suggest that the questionnaire is a reasonable way to assess registered nurses’ perceptions of use of personal communications devices. However, more work is needed to test it on a larger sample.

The test-retest results were found to be only fair to moderate [33]. This can be attributed to the following two reasons: (1) a small test-retest sample increases the statistical error and results in a low Cohen’s kappa value, and (2) many questions relate to experiences and attitudes regarding personal communication device issues, which may change during a 1-week period. The changes within the 1 SD range from test to retest on the Likert scale indicate that even with the small sample, the stability is acceptable.

No specific questions had more than 7% missing data, and the percentage of missing data did not increase toward the end of the surveys, indicating that respondents did not consider the questionnaire to be too lengthy.

Unknown factors could confound the differences identified in this study. Despite the small sample size, significant differences were found in specific areas where a focus on utilization issues and guidelines for use on nursing units would be expected to have an impact. Although none of the units where the respondents were working had formal policies on personal communication device use that were enforced, it is considered likely that the guidelines and general focus on utilization issues could be major contributors to the differences among registered nurses based on the surveys. As such, it is plausible that our questionnaire will detect differences among a larger sample of surveyed nurses.

With respect to construct validity, most of the questions were direct (ie, they were asked directly about the construct they wished to measure). This increases the chance of the instrument actually measuring the desired construct. The comments added by the respondents indicated that the questions had been understood and responded to as expected. According to Terwee et al [25], conducting surveys among health care professionals involves a fairly homogenous group to whom personal communication devices are well known. This also increases the chances of the instrument measuring the desired concepts.

### Validation Concerns

Tests for internal consistency and criterion validity may also be conducted during the validation of questionnaire instruments [22,25]. However, this was not applicable for the present instrument because there is no objective standard against which to test the correlation.

Another weakness of the validation study was the small sample size of the study. Because measuring the relation between nonresponse and the accuracy of a survey statistic is complex and expensive, few rigorously designed studies provided empirical evidence to document the consequences of lower response rates, until recently. Keeter et al[34] compared the results of a 5-day survey employing the Pew Research Center’s usual methodology (with a 25% response rate) with those from a more rigorous survey conducted over a much longer field period, achieving a higher response rate of 50%. In 77 of 84 (91%) comparisons, the two surveys yielded results that were statistically indistinguishable. Among the items that manifested significant differences across the two surveys, the differences in proportions of people giving a particular answer ranged from 4 percentage points to 8 percentage points. Holbrook et al [35] assessed whether lower response rates are associated with less unweighted demographic representativeness of a sample. By examining the results of 81 national surveys with response rates varying from 5% to 54%, they found that surveys with much lower response rates were only minimally less accurate. Nevertheless, the small initial sample size did both increase the statistical error in the test-retest analysis and prevent extensive subanalyses in the comparison between the two tests. Further testing with a much larger sample would be necessary to overcome this limitation.

The low Cronbach alpha values for Group 2 questions may indicate that the assumptions underlying the Cronbach alpha calculation have been violated for this group of questions. In other words, the questions may not all be measuring the same underlying dimension, or the coding for some questions may need to be reversed. However, examining this set of questions in a variety of alternative ways (eg, not inverting the scale for negative questions) did not consistently increase the alpha values. Some of these modifications increased the estimate of reliability for Week 1 for at least some subsets of questions, but generally at the expense of the reliability for Week 2 or other question subsets. These results indicate that it may not be logical to consider the questions in this group as representative of a single underlying dimension.

Alternatively, the low Cronbach alpha score associated with these questions may reflect a tendency among respondents to underrecognize their own distraction, or a discomfort in reporting self-use. Nurses’ reported observations of other registered nurses’ use of personal communication devices were higher than the self-reported results. This discrepancy may be because nurses did not believe that they were distracted. In a study of drivers, Lesch and Hancock [36] found that drivers did not recognize that their driving ability worsened when they were using a cell phone. Strayer et al [37] also noted that drivers using cell phones described other drivers with cell phones as driving inconsistently, but believed that their own performance was unaffected, even when results showed otherwise. This led the researchers to believe that cell phone use may make drivers unaware of their own attention deficits. In the pilot survey, the participants’ ambivalence about answering these difficult questions may therefore have been responsible for the low Cronbach alpha scores, but the importance of the answers argues for keeping the questions on the survey. The self-reported rate versus reporting of other nurses’ behavior will provide a useful comparison in future studies.

The Cronbach alpha for the utilization scale (Group 1) was higher than that for the performance scale (Group 2). This may be because it is easier to identify specific behaviors associated with the use of personal communication devices than attitudes and values associated with job performance. Although the items in the performance scale appear related to one another and to the concept of behavior associated with job performance, they had low inter-item correlations. This indicates that there was some acceptable commonality among some of the items. Despite these acceptable correlations, the alpha for the factor was lower than desirable, implying that the items may not in fact belong together. These results may reflect the complex role of performance in nursing and the fact that although job performance is an important concept, it may not be possible to encapsulate it easily in a question.

### Limitations

Several limitations of the present study should be acknowledged. Self-selection bias affects any survey that allows respondents to decide whether to participate. To mitigate this potential problem, we compared the characteristics of the respondents in our study with those of California-based registered nurses and found that the respondents in our study were not systematically different from those of the state’s average registered nurse in terms of gender, age, race/ethnicity, job title, and experience with personal communication devices. However, this is a potential problem should the survey be used elsewhere and more widely, and similar tests will need to be carried out to ensure there is no self-selection bias. Discussions during the validation process suggested that some responses might be skewed by the fact that respondents may prefer not to admit that they made a medical error or that their performance had suffered as a result of their use of personal communications devices. For this reason, questions were rewritten for the survey questionnaire to ask if respondents had seen other registered nurses making a medical mistake because of distraction caused by their personal communication device or if they knew of a major medical error that had been caused by a registered nurse who was similarly distracted. By reducing self-reporting in this manner, it was hoped that underreporting of “bad” behavior resulting from said distraction would be lessened.

In addition, the validation process was conducted in the United States. As the use of personal communication device varies in different cultures and across professions, the questionnaire may not be easily translated to other countries or professions. However, we believe that it will be useful as a model to develop different national questionnaires.

### Next Steps

The work set out in this paper is very much a preliminary stage in a much larger piece of work. We have reported on the initial development and pilot testing of an online survey to assess registered nurses’ views on the use of personal communication devices. The next step is to make some specific changes, and then use the survey in a proposed study of California registered nurse licensees. The following three specific changes have been proposed for the survey. First, the definition of “personal communication device” needs to be clarified. There may have been some confusion about whether a hospital-owned device such as an electronic device should be considered as a personal communication device. When using the survey with a larger group, we plan to include a definition of “personal communication device” at the beginning, to make clear that it is the one that is owned by the nurse, and used for personal business or family matters, as compared with professional or work-related activities.

Second, the type of unit that the registered nurse respondent is working on may have some sort of bearing on the use of personal communication devices, for example, in terms of the levels of concentration required of nurses.

Finally, the length of time during use as well as the frequency of device use should also be kept in consideration.

The results of the proposed study will provide data concerning the use of personal communication devices on nursing units and the opinion of nurses on the potential impact, both positive and negative, of personal communication devices on patient safety. These data may inform hospital policies and practices concerning the use of personal communication devices on hospital units. An acceptable hospital policy needs to be based on evidence and should aim to create a reasonable balance between work-related and personal use.

### Suggestions for Future Research

In future, it may be helpful to expand the survey to include additional questions to identify times and places where important information is transferred or interaction occurs, such as during attending rounds, morning huddle, or at the bedside. Mitigation techniques could also be surveyed to better understand how respondents handle distraction caused by their or others’ personal communication devices.

### Conclusions

Knowledge about personal communication practices in hospital units is important to improve clinical practice and patient care, and a valid questionnaire assists in detecting the issues that need to be improved. Our results describe the development of one possible online questionnaire to establish registered nurses’ views concerning the impact of personal communication devices on hospital units. Statistical tests showed both good and moderate areas of validity of the questionnaire. The psychometrics performed on the survey questionnaire indicated that the survey has the potential to be a useful tool to assess hospital nurses’ perceptions of personal communication device use in the workplace, and we plan to use it to carry out a wider survey of nurses in California in the future.

## References

[1] Baumgart DC (2011). Smartphones in clinical practice, medical education, and research. Arch Intern Med.

[2] Divall P, Camosso-Stefinovic J, Baker R (2013). The use of personal digital assistants in clinical decision making by health care professionals: a systematic review. Health Informatics J.

[3] Florczak B, Scheurich A, Croghan J, Sheridan P, Kurtz D, McGill W, McClain B (2012). An observational study to assess an electronic point-of-care wound documentation and reporting system regarding user satisfaction and potential for improved care. Ostomy Wound Manage.

[4] Shepherd JD, Badger-Brown KM, Legassic MS, Walia S, Wolfe DL (2012). SCI-U: e-learning for patient education in spinal cord injury rehabilitation. J Spinal Cord Med.

[5] Dala-Ali BM, Lloyd MA, Al-Abed Y (2011). The uses of the iPhone for surgeons. Surgeon.

[6] Jensen Ang WJ, Hopkins ME, Partridge R, Hennessey I, Brennan PM, Fouyas I, Hughes MA (2013). Validating the use of smartphone-based accelerometers for performance assessment in a simulated neurosurgical task. Neurosurgery.

[7] Sohn W, Shreim S, Yoon R, Huynh VB, Dash A, Clayman R, Lee HJ (2013). Endockscope: using mobile technology to create global point of service endoscopy. J Endourol.

[8] Zuo KJ, Guo D, Rao J (2013). Mobile teledermatology: a promising future in clinical practice. J Cutan Med Surg.

[9] ECRI Institute (2012). Top 10 technology hazards for 2013. Health Devices.

[10] JACHO (2002). Preventing ventilator-related deaths and injuries. Sentinel Event Alert.

[11] Stevenson JG (2005). Medication errors: experience of the United States pharmacopeia (USP). Jt Comm J Qual Saf.

[12] American College of Surgeons (2008). Statement on the use of cell phones in operating room. Bull Am Coll Surg.

[13] Hopkinson SG, Jennings BM (2013). Interruptions during nurses' work: a state-of-the-science review. Res Nurs Health.

[14] Payne KB, Wharrad H, Watts K (2012). Smartphone and medical related App use among medical students and junior doctors in the United Kingdom (UK): a regional survey. BMC Med Inform Decis Mak.

[15] Naumann RB, Dellinger AM (2011). Mobile device use while driving–United States and seven European countries. MMWR.

[16] Hoff J, Grell J, Lohrman N, Stehly C, Stoltzfus J, Wainwright G, Hoff WS (2013). Distracted driving and implications for injury prevention in adults. J Trauma Nurs.

[17] Schweizer TA, Kan K, Hung Y, Tam F, Naglie G, Graham SJ (2013). Brain activity during driving with distraction: an immersive fMRI study. Front Hum Neurosci.

[18] Weigl M, Müller A, Vincent C, Angerer P, Sevdalis N (2012). The association of workflow interruptions and hospital doctors' workload: a prospective observational study. BMJ Qual Saf.

[19] Gill PS, Kamath A, Gill TS (2012). Distraction: an assessment of smartphone usage in health care work settings. Risk Manag Healthc Policy.

[20] Smith T, Darling E, Searles B (2011). 2010 Survey on cell phone use while performing cardiopulmonary bypass. Perfusion.

[21] Black E, Light J, Paradise Black N, Thompson L (2013). Online social network use by health care providers in a high traffic patient care environment. J Med Internet Res.

[22] DeVellis RF (2012). Guidelines in scale development. Scale Development: Theory and Applications (Applied Social Research Methods). Volume 26. 3rd edition.

[23] Bowling A (2009). Research methods in health: investigating health and health services. 3rd edition.

[24] Moulton PL, Wiebusch PL, Cleary BL, Brunell ML, Napier DF, Bienemy C, LeVasseur SA, Cimiotti JP (2012). Toward standardization (part 2): national nursing minimum data sets consensus building and implementation status. Policy Polit Nurs Pract.

[25] Terwee CB, Bot SD, de Boer MR, van der Windt DA, Knol DL, Dekker J, Bouter LM, de Vet HC (2007). Quality criteria were proposed for measurement properties of health status questionnaires. J Clin Epidemiol.

[26] Dillman DA, Smyth JD, Christian LM (2009). Mail and Internet surveys: the tailored design method. Internet, Mail, and Mixed-Mode Surveys: The Tailored Design Method. 2nd edition.

[27] Allen MJ, Yen WM (2002). Introduction to measurement theory. Introduction to Measurement Theory.

[28] Julius SA (2005). Sample size of 12 per group rule of thumb for a pilot study. Pharm Stat.

[29] Kvale S, Brinkmann S (2009). InterViews: Learning the Craft of Qualitative Research Interviewing.

[30] Graneheim UH, Lundman B (2004). Qualitative content analysis in nursing research: concepts, procedures and measures to achieve trustworthiness. Nurse Educ Today.

[31] Tavakol M, Dennick R (2011). Making sense of Cronbach's alpha. Int J Med Educ.

[32] Allison PD (2002). Missing data.

[33] Streiner DL, Norman GR (2008). Health Measurement Scales: A Practical Guide to Their Development and Use. 4th edition.

[34] Keeter S, Kennedy C, Dimock M, Best J, Craighill P (2006). Gauging the impact of growing nonresponse on estimates from a National RDD Telephone Survey. Public Opin Q.

[35] Lepkowski JM, Tucker C, Leeuw EDD, Japec L, Lavrakas P, Link M, Sangster RL, Lepkowski JM, Tucker NC, Brick M, DeLeeuw ED, Japec L, Lavrakas PJ, Link ML, Sanger RL (2008). The causes and consequences of response rates in surveys by the news media and government contractor survey research firms. Advances in Telephone Survey Methodology.

[36] Lesch MF, Hancock PA (2003). Driving performance during concurrent cell-phone use: are drivers award of their performance decrements?. Accid Anal Prev.

[37] Strayer DL, Drews FA, Johnston WA (2003). Cell phone-induced failures of visual attention during simulated driving. J Exp Psychol Appl.

